# Dietary Crude Protein and Lysine Levels Affect Meat Quality and Myofiber Characteristic of Slow-Growing Chicken

**DOI:** 10.3390/ani14142068

**Published:** 2024-07-15

**Authors:** Cheng Chang, Weiyu Zhao, Qianqian Zhang, Xuan Wang, Jian Zhang, Zhixun Yan, Jing Cao, Huagui Liu, Ailian Geng

**Affiliations:** Institute of Animal Husbandry and Veterinary Medicine, Beijing Academy of Agriculture and Forestry Sciences, Beijing 100097, China; changeng02@163.com (C.C.); zhaoweiyu105@163.com (W.Z.); zqdyxn@163.com (Q.Z.); 15711469675@163.com (X.W.); zjcau@126.com (J.Z.); yanzhixun2008@sina.com (Z.Y.); caojing2046555@163.com (J.C.)

**Keywords:** crude protein, lysine, meat quality, myofiber, growing chicken

## Abstract

**Simple Summary:**

Nutritional factors are crucial to performance and meat quality. We investigated the effects of dietary CP and lysine levels on growth performance, slaughter performance, meat quality, and myofiber characteristics in slow-growing chicken and found that 18.0% CP increased BWG and shear force and had the lowest myofiber density and the largest myofiber diameter. Dietary lysine level affected myofiber diameter. Reducing dietary CP level and adding appropriate lysine can reduce myofiber diameter and increase perimysium thickness and endomysium thickness, therefore reducing shear force and improving meat tenderness.

**Abstract:**

This study aimed to investigate the effects of dietary crude protein (CP) and lysine levels on growth performance, slaughter performance, meat quality, and myofiber characteristics of slow-growing chicken. A 3 × 3 factorial experiment was arranged, and the chickens were fed with 3 levels of dietary CP (16.0%, 17.0%, 18.0%) and 3 levels of dietary lysine (0.69%, 0.84%, 0.99%). A total of 540 8-week-old Beijing-You Chicken (BYC) female growing chickens were randomly allocated to 9 groups, 5 replicates per group, and 12 chickens per replicate. The birds were randomly allocated to one of the 9 experimental diets. Growth performance, slaughter performance, meat quality, and myofiber characteristics were determined at 16 weeks of age. The results showed that dietary CP level and the interaction of dietary CP and lysine levels affected average feed intake (AFI) (*p* < 0.05). The AFI in the 16.0% CP and 17.0% CP groups was higher than in the 18.0% CP group (*p* < 0.05). Dietary CP levels significantly affected body weight gain (BWG) (*p* < 0.05) at 9 to 16 weeks. The 18.0% CP group had the highest BWG (93.99 g). Dietary CP levels affected the percentage of leg muscle yield, and the percentage of leg muscle yield of the 16.0% CP group was significantly lower than that in the other two groups (*p* < 0.05). Dietary CP and lysine levels alone and their interactions did not affect pH_24h_, drip loss, and cooking loss of breast muscle (*p* > 0.05). The shear force of the 18.0% CP group (29.55 N) was higher than that in the other two groups (*p* < 0.01). Dietary CP level affected myofiber characteristic (*p* < 0.01), with the lowest myofiber density (846.35 p·mm^−2^) and the largest myofiber diameter (30.92 μm) at 18.0% CP level. Dietary lysine level affected myofiber diameter, endomysium thickness, perimysium thickness (*p* < 0.01), with the largest myofiber diameter (29.29 μm) obtained at 0.84% lysine level, the largest endomysium thickness (4.58 μm) at 0.69% lysine level, and the largest perimysium thickness (9.26 μm) at 0.99% lysine level. Myofiber density was negatively correlated with myofiber diameter and endomysium thickness (R = −0.883, R = −0.523, *p* < 0.01); perimysium thickness had a significant negative correlation with shear force (R = −0.682, *p* < 0.05). Therefore, reducing dietary CP level and adding appropriate lysine can reduce myofiber diameter and increase perimysium thickness, reducing shear force and improving meat tenderness. A high lysine level (0.99%) in the low-CP (16.0%) diet can improve meat tenderness by regulating the myofiber characteristic without affecting production performance.

## 1. Introduction

Chicken is currently the most consumed meat globally due to its availability and high nutritional value. With the improvement of people’s living standards, people began to pursue meat with high quality and high nutritional value. In recent decades, genetic selection for body weight, growth rate, and feed conversion rate have contributed to a prominent increase in production efficiency and meat yield; however, there has been a parallel decrease in meat quality. How to regulate and obtain high-quality chicken products through nutritional regulation while maintaining performance is an important topic.

The modern concept of meat quality is composed of its edible quality (meat color, tenderness, flavor, etc.), nutritional quality (content of amino acids and fatty acids, etc.), technical quality (pH, etc.), health quality (drug residues, heavy metal ion concentration, etc.) and humanistic quality (cultural patterns and breeding patterns) [1]. The edible quality mainly depends on the change in myofiber characteristics, which generally include myofiber type, myofiber diameter, myofiber density, perimysium thickness, endomysium thickness, etc. The morphological and biochemical properties of myofibers play an important role in determining the physicochemical properties of muscle [2,3].

Nutritional factors are crucial to meat quality. The protein required for growth and production is derived from dietary crude protein (CP). Dietary CP level has a profound effect on broiler growth, health, and meat quality [4,5,6]. Appropriate CP levels can improve the performance of broilers. A low CP level will lead to growth retardance and reduced meat yield, while a high CP level will not only increase the cost but also result in excessive nitrogen emission into the environment and cause pollution [7,8]. Lysine is a limiting amino acid and one of the essential amino acids for growth, development, and reproduction in animals. Dietary lysine supplementation improves the growth and performance of poultry [9,10,11], affects the synthesis and hydrolysis of protein in broilers, and lysine deficiency results in decreased protein deposition [12,13,14,15].

With the introduction of an ideal protein model, amino acid supplementation in diets with low CP levels can improve performance and productivity and reduce nitrogen excretion [16]. A low-protein diet can accurately meet the nutritional requirements of poultry by supplementing essential amino acids while saving feed costs and reducing nitrogen excretion. Urdaneta-Rincon found that dietary lysine levels significantly affected muscle synthesis rate when the protein level was 170 g/kg and 210 g/kg [17]. An accurate balance of dietary lysine and CP levels can significantly improve the growth rate and muscle synthesis rate of broilers [18,19,20]. Therefore, precision nutrition studies based on essential amino acids such as lysine will lay a foundation for the formulation of low-CP diets [20,21,22,23,24], which can reduce environmental pollution while maintaining the production performance of chickens, improving meat quality, and obtaining better chicken products. This study aimed to investigate the effects of dietary CP and lysine levels alone and their interactions on growth performance, slaughter performance, meat quality, and myofiber characteristics in Beijing-You Chicken (BYC), which is a slow-growing and dual-purpose chicken [25], usually marketed at 110~120 days of age, with an average weight of 1.4–1.6 kg [26].

## 2. Materials and Methods

### 2.1. Experimental Design and Birds

The experiment was conducted at a commercial BYC farm in the Shunyi district of Beijing. A total of 540 8-week-old BYC female growing chickens with an average body weight of 566.45 g were randomly allocated to 9 groups, 5 replicates per group, 12 chickens per replicate (one replicate, three cages, four birds a cage). The birds were randomly allocated to one of the 9 experimental diets, and the diets were provided in powder form. The main nutritional parameters of the chicken at the first 7 weeks of life were 12.12 MJ/kg metabolizable energy, 19.0% CP, 0.98% lysine, 0.75% methionine and cystine, 0.85% calcium, and 0.45 non-phytate phosphorous. The composition and nutritional levels of this experimental diet are seen in Table 1. The CP and lysine levels were adjusted up and down according to the recommended nutrient levels of 17.0% CP and 0.84% lysine in the “Technical Code of Practice of Feeding and Management of Beijing-You Chicken” [27]. Dietary CP and amino acids were measured according to national standards [28,29].

### 2.2. Feeding and Management

The chickens were reared in A-type cages. The cage is used for the chicks and growing chickens. The length is 65 cm, the height is 40 cm, and the depth is 50 cm. The replicate cages were evenly distributed in the house. The chickens were fed ad libitum, had free access to water, and were immunized according to “The vaccination program of commercial Beijing-You Chicken” of the farm. The temperature, relative humidity, lighting conditions, and other management were manipulated according to the “Technical Code of Practice of Feeding and Management of Beijing-You Chicken” [27]. Use energy-saving fluorescent lamps. The bulb is 2 m above the ground, and the average light intensity is 10 lx. The pre-trial lasted for 1 week, and the formal experimental period was from 9 to 16 weeks of age.

### 2.3. Measurement

Feed intake and body weight of chickens in each replicate cage were recorded every week, and weekly average feed intake (AFI), body weight gain (BWG), feed gain ratio (F/G), and mortality rate were calculated from 9 to 16 weeks of age.

At the end of 16 weeks, five birds from each group were randomly chosen and euthanized by cervical dislocation after 12 h of feed deprivation. The dressed weight, half-eviscerated weight with giblet, eviscerated weight, breast muscle yield and leg muscle yield were determined. The following indicators were calculated according to “Performance terminology and measurements for poultry” [30]: dressed percentage, percentage of half-eviscerated yield with giblet, and percentage of eviscerated yield were based on live weight before slaughter, while the percentage of breast muscle yield and percentage of leg muscle yield was based on eviscerated yield. One side of the breast muscle for meat quality measurement included pH_24h_, shear force, drip loss, and cooking loss. At 24 h after slaughter, the pH_24h_ of the breast muscle was measured using a portable pH meter (Testo 206, Testo, Lenzkirch, Germany), which was calibrated using buffers (PH:4.01, 7.01) to ensure accuracy before the measurements. The measurement of drip loss, cooking loss, and shear force were referred to Chen et al. [31]

Another side of the breast muscle (pectoralis major), a sample of about 2 cm × 0.5 cm × 0.5 cm was cut in the middle along the direction of the myofiber, then dehydrated and embedded in paraffin and the sections were hematoxylin-eosin stained. Subsequently, the samples were photographed with upright microscopy (Model: BX51, Olympus, Japan), and the images were processed by CellSens Standard Software (1.6, Olympus, Tokyo, Japan). Six photographs were taken for each sample. Myofiber diameter, the number of fibers in the muscle bundle, endomysium thickness, and perimysium thickness were measured and calculated using ImageJ software (1.52p, National Institutes of Health, Bethesda, MD, USA).

Myofiber diameter: measure the area (S) of intact myofibers in all sections and calculate the myofiber diameter (L): L = $2*\sqrt{S/\pi }$.

Myofiber density: count the number of myofibers in all sections (N) and calculate the density of myofiber (D): D = N/S.

Number of fibers in the muscle bundle: select five muscle bundles in each section and use the count function in Image J software to count the number of myofibers in each muscle bundle.

Endomysium thickness: measure the distance between two adjacent myofibers within the muscle bundle and measure 100 values in each section.

Perimysium thickness: measure the distance between two adjacent muscle bundles and measure 100 values in each section.

### 2.4. Statistical Analyses

Data were analyzed using the General Linear Model procedure in SPSS 25.0 software for Windows (SPSS Inc. Chicago, IL, USA). The nutritional levels (CP% and lysine%) were analyzed as the main effects, and Duncan’s Test was used for multiple comparisons (*p* < 0.05). The growth performance, slaughter performance, meat quality, and myofiber characteristics data were analyzed using the PROC MIXED procedure of SPSS 25.0 software for Windows (SPSS Inc., Chicago, IL, USA). The effects of dietary CP level (16.0%, 17.0%, 18.0%) and lysine level (0.69%, 0.84%, 0.99%) were analyzed as a 3 × 3 factorial design. The model included CP level, lysine level and their interactions as fixed effects and replication as a random effect. Duncan’s Test was used to make multiple comparisons and identify significant differences.

The percentage was arcsine transformed before the normality test, with *p* < 0.01 as very significant, 0.01 < *p* < 0.05 as significant, and 0.05 < *p* < 0.10 as having a significant trend. The growth performance, slaughter performance, meat quality, and myofiber characteristic data were expressed as mean and standard deviation. The relationship between myofiber characteristics and meat quality indicators was assessed using Pearson’s correlation coefficient.

## 3. Results

### 3.1. Growth Performance

Table 2 showed that dietary CP level and the interaction of dietary CP and lysine levels affected AFI (*p* < 0.05). The AFI in the 16.0% CP and 17.0% CP groups was higher than in the 18.0% CP group (*p* < 0.05). The AFI in the 16.0% CP and 0.69% lysine group (480.80 g) and 17.0% CP and 0.99% lysine group (480.25 g) were higher than those of the other groups (*p* < 0.05). Dietary CP levels significantly affected BWG at 9~16 weeks of age (*p* < 0.05). The BWG increased significantly with the increase in dietary CP levels. The 18.0% CP group had the highest BWG (93.99 g). The mortality rate in the 17.0% CP group has an increasing trend (*p* = 0.086).

### 3.2. Slaughter Performance

Table 3 showed that dietary CP and lysine levels alone and their interactions had no significant effects on dressed percentage, percentage of half-eviscerated weight with giblet, percentage of eviscerated weight, and percentage of breast muscle yield (*p* > 0.05). Dietary CP levels affected the percentage of breast muscle yield, and the percentage of breast muscle yield of the 16.0% CP group was significantly lower than that of the other two groups (*p* < 0.01).

### 3.3. Meat Quality

Table 4 showed that dietary CP and lysine levels alone and their interactions did not affect pH_24h_, drip loss, and cooking loss of chicken breast meat (*p* > 0.05). Dietary CP levels significantly affected shear force (*p* < 0.01). The shear force of the 18.0% CP group (29.55 N) was significantly higher than that in the other two groups (*p* < 0.01).

### 3.4. Myofiber Characteristics

**Figure 1:**
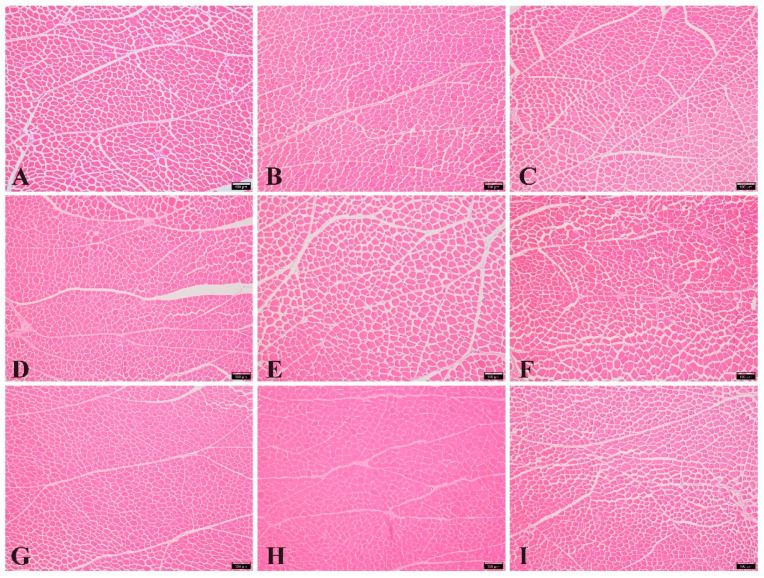
The myofiber characteristics of slow-growing chickens at 16 weeks of age with different dietary CP and lysine levels. (**A**) CP:16.0%, lysine:0.69%; (**B**) CP:16.0%, lysine:0.84%; (**C**) CP:16.0%, lysine:0.99%; (**D**) CP:17.0%, lysine:0.69%; (**E**) CP:17.0%, lysine:0.84%; (**F**) CP:17.0%, lysine:0.99%; (**G**) CP:18.0%, lysine:0.69%; (**H**) CP:18.0%, lysine:0.84%; (**I**) CP:18.0%, lysine:0.99%. CP = crude protein. Hematoxylin and eosin (H&E) staining, magnification: 100×.

As shown in Figure 1, the number of myofibers under the same microscope vision looks different at different dietary CP and lysine levels. The number of myofibers in group H (CP: 18.0%, lysine: 0.84%) appears to be large, and the endomysium thickness is relatively small, while the number of myofibers in group E (CP: 17.0%, lysine: 0.84%) appears to be small and the endomysium thickness is relatively large. It seems that a higher CP level affects the number of myofibers and endomysium thicknesses.

Table 5 showed that dietary CP and lysine levels alone and in interaction had significant effects on myofiber characteristics (*p* < 0.05). Dietary CP level significantly affected myofiber density, myofiber diameter, endomysium thickness and perimysium thickness (*p* < 0.01), the myofiber density of the 16.0% CP group (991.70 p·mm^−2^) was higher than that in the other two groups (*p* < 0.01), and the myofiber diameter of the 18.0% CP group (30.92 μm) was higher than that in the other two groups (*p* < 0.01), Both endomysium thickness and perimysium thickness of the 17.0% CP group (4.83 μm, 9.97 μm) was higher than that in the other two groups (*p* < 0.01). Dietary lysine level significantly affected myofiber diameter, endomysium thickness, and perimysium thickness (*p* < 0.01), with the myofiber diameter of the 0.84% lysine group (29.29 μm) being higher than that in the other two groups (*p* < 0.01). Both endomysium thickness and perimysium thickness of the 0.84% lysine group (4.09 μm, 6.91 μm) were lower than that in the other two groups (*p* < 0.01). The myofiber density was the highest, and the myofiber diameter was the lowest in the group, with 16.0% CP and 0.99% lysine.

The correlation between myofiber characteristics and meat quality indicators is shown in Table 6. Myofiber density was negatively correlated with myofiber diameter and endomysium thickness (*R* = −0.883, *R* = −0.523, *p* < 0.01); The perimysium thickness was positively correlated with endomysium thickness (*R* = 0.764, *p* < 0.05) and negatively correlated with shear force (*R* = −0.682, *p* < 0.05); Shear force was positively correlated with cooking loss (*R* = 0.380, *p* < 0.05); Myofiber diameter was positively correlated with endomysium thickness (*R* = 0.448, *p* < 0.01) and negatively correlated with perimysium thickness (*R* = −0.437, *p* < 0.05); Cooking loss was positively correlated with drip loss (*R* = 0.546, *p* < 0.01) and negatively correlated with pH_24h_ (*R* = −0.834, *p* < 0.01).

## 4. Discussion

Dietary CP is one of the most important nutritional compositions and one of the main indicators for evaluating the nutritional value of feed. As an essential amino acid, lysine is important for growth performance, and within a certain range, the increase of lysine level can increase the growth performance of poultry [32,33]. Notably, the effects of dietary CP and lysine levels on performance have focused on feed conversion ratio, growth rate, and meat yield [34,35,36,37], and relatively few studies focused on meat quality [15]. Reducing dietary CP levels can increase abdominal fat accumulation in broilers, thus improving meat color [38,39]. Decreasing dietary CP levels reduced the meat yield of broilers [4,40]. Increasing dietary lysine levels increase the pH and decrease drip loss in breast meat [41], and high lysine levels increase meat yield in broilers [42]. Chodová et al. indicated greater differences in meat quality owing to genotype than to sex or dietary protein level. The low-protein diet was associated with changes in meat quality parameters, including increased drip loss and muscle fiber area [43].

Dietary CP levels have a significant impact on growth performance, with insufficient amounts of CP levels leading to growth retardation [4,44]. Law et al. found that the growth performance of broilers gradually deteriorated with the decrease in dietary CP level [45]. Amino acid levels in low-protein diets have important effects on the growth performance of broilers [46,47]. Reducing CP level by 2% and supplementing with amino acids resulted in better growth performance and F/G in the low dietary CP group [48]. In the present study, there was a significant increase in BWG with the increase in dietary CP level, and the BWG was the highest in the 18.0% CP group (93.99 g), but F/G was not affected. We found that lysine supplementation at a certain dietary CP level could improve growth performance, and feed intake decreased significantly when dietary CP levels increased, inconsistent with the previous results [49,50,51]. A human study has shown that high-protein diets can increase appetite hormones, increase satiety, and reduce appetite, such as glucagon-like peptide-1 (GLP-1), cholecystokinin (CCK), and peptide tyrosine-tyrosine (PYY) [52], which applies to the results of this study.

No significant effect of dietary CP and lysine levels on slaughter performance was found in this study, which is similar to previous studies [40,53]. Van Harn et al. concluded that appropriate adjustment and supplementation of some essential amino acids, along with changes in dietary protein levels, would not adversely affect the slaughter performance of broilers [40]. Ospina-Rojas et al. showed that the addition of glycine and arginine to a low-protein diet of broilers, along with supplementation with essential amino acids such as valine, isoleucine, and lysine, maintained dressed weight [53]. The percentage of breast muscle yield increased with the increase in dietary CP level, which may be related to the effects of dietary CP level on the growth, development, and protein deposition of broilers [54]. Muscle, as one of the major sites of protein deposition, is more sensitive to dietary CP level, which is consistent with previous reports [20,26,35,54,55]. Mousa et al. found that increasing dietary CP level and lysine level during growth could significantly increase breast muscle content [56]. The lysine is mainly involved in biochemical reactions required for early growth in broilers [57], which plays a key role in muscle growth and protein synthesis [10]. In this study, the percentage of breast muscle yield in the group with 17.0% CP and 0.84% lysine was the highest, indicating that increasing CP levels and adding appropriate lysine could increase meat yield, which is similar to the other studies [58,59].

Myofiber diameter and myofiber density are important indicators of meat tenderness. Myofiber diameter could be reduced by a low-protein diet [60,61]. In the present study, the shear force increased with increasing dietary CP level, and myofiber density, myofiber diameter, endomysium thickness, and perimysium thickness were affected by dietary CP level. An 18.0% CP level caused myofibers to thicken and reduce myofiber density, while thickening myofibers led to increased toughness and stiffness. It may be related to the fact that dietary CP level controls muscle growth by regulating the expression of MSTN and MRF genes [62]. It is also possible that dietary CP levels affect the synthesis of proteolytic enzymes [63], such as calcium-dependent proteases, one of the key factors affecting meat tenderness. Enhanced activities of calcium-dependent proteases resulted in muscle rejuvenation [64,65]. The lysine significantly affects the protein synthesis rate, hydrolysis, and deposition in broiler muscle [66] and has a significant effect on myofiber diameter, with the smallest myofiber diameter at a lysine level of 0.99%.

Zhai et al. found that the lysine levels in diet supplementation reduced meat shear force [67]. The lysine levels promoted optimal muscle growth by upregulating MyoD mRNA expression and inhibiting MSTN expression [68], and appropriate levels of dietary lysine could improve meat quality by controlling myofiber diameter [69]. The interaction of dietary CP and lysine levels significantly affected myofiber diameter. The myofiber diameter in the 16.0% CP and 0.99% lysine group was the smallest (24.29 μm), indicating that increasing lysine level could regulate myofiber diameter, reduce shear force, and increase meat tenderness under low CP level.

Both the endomysium and perimysium belong to the connective tissue of muscles. Generally, more water storage space is supported by richer connective tissue, ensuring greater water-holding capacity and more tender muscles [70]. In this study, dietary CP and lysine levels alone and their interaction significantly affected the endomysium thickness and perimysium thickness. The maximum values were obtained at 17.0% CP and 0.99% lysine levels (5.29 μm, 12.56 μm). Collagen is a major component of muscle connective tissue [71], and appropriate dietary CP levels could increase LARP6a expression through IGF-1, PI3K, Akt, and p70S6K signaling pathways, which increases collagen synthesis [72,73,74]. Appropriate lysine level can also regulate collagen synthesis and deposition in muscle through the activation of key proteins in the mTORC1 signaling pathway [75,76], which helps explain the highest content of connective tissue in the 0.99% lysine group.

The endomysium thickness was positively correlated with myofiber diameter and perimysium thickness, indicating that muscle connective tissue is uniformly distributed inside and outside the muscle bundles, and the thicker the endomysium, the larger the myofiber diameter. The pH_24h_ was significantly negatively correlated with drip loss and cooking loss, which was similar to the study of Kim et al. [77]. Muscle exudates are mainly water, proteins, peptides, etc., and the cause of such exudation is often closely related to oxidation and denaturation of muscle proteins [78,79], which is also one of the main reasons for muscle pH changes. In this study, the shear force was positively correlated with cooking loss and negatively correlated with the endomysium thickness, indicating that the richer the connective tissue in the muscle, the stronger the water-holding capacity and the more tender the meat, which is consistent with Srikanchai et al. [70] and Olsson et al. [80]. The content and nature of collagen is one of the important factors affecting meat tenderness [81,82], which also supported the results of this study.

## 5. Conclusions

Dietary CP level affected AFI and BWG. The 18.0% CP group had the highest BWG at 9 to 16 weeks of age. Dietary CP levels affected the percentage of breast muscle yield. The percentage of breast muscle yield in the 18.0% CP group was significantly higher than that in the other two groups.

Reducing dietary CP level and adding appropriate lysine can reduce myofiber diameter and increase perimysium thickness and endomysium thickness, therefore reducing shear force and improving tenderness.

Dietary lysine levels affect myofiber diameter, endomysium thickness, and perimysium thickness of slow-growing chicken. High lysine (0.99%) in the low-CP (16.0%) diet can improve meat tenderness by regulating the myofiber characteristic without affecting the production performance, which will benefit the application of a low-CP diet in the poultry industry in the future.

## Figures and Tables

**Table 1:** Composition and nutritional levels of the experimental diets (air-dry basis).

CP, %	16.0	16.0	16.0	17.0	17.0	17.0	18.0	18.0	18.0
Lysine, %	0.69	0.84	0.99	0.69	0.84	0.99	0.69	0.84	0.99
Ingredients, %	1	2	3	4	5	6	7	8	9
Corn	64.05	65.30	65.80	58.20	62.60	63.10	56.37	59.90	60.50
Wheat bran	12.80	9.80	9.60	16.50	11.00	10.90	17.00	12.20	12.00
Soybean meal	14.00	19.00	19.00	13.00	18.10	18.00	12.00	17.10	17.10
Corn gluten meal	4.15	0.90	0.60	6.40	3.30	3.00	9.00	5.80	5.40
Soybean oil	0.00	0.00	0.00	0.90	0.00	0.00	0.63	0.00	0.00
Vitamin premix ^1^	0.03	0.03	0.03	0.03	0.03	0.03	0.03	0.03	0.03
50% Choline chloride	0.08	0.08	0.08	0.08	0.08	0.08	0.08	0.08	0.08
DL-Methionine	0.12	0.14	0.15	0.07	0.09	0.10	0.02	0.04	0.05
70% Lysine	0.00	0.09	0.37	0.00	0.09	0.37	0.00	0.09	0.37
98% Threonine	0.08	0.06	0.07	0.04	0.03	0.04	0.01	0.00	0.00
Mineral premix ^2^	0.15	0.15	0.15	0.15	0.15	0.15	0.15	0.15	0.15
Zeolite powder	0.94	0.88	0.58	1.03	0.96	0.66	1.11	1.04	0.75
Limestone	1.67	1.64	1.64	1.67	1.64	1.64	1.67	1.64	1.64
Calcium bisphosphate	1.23	1.23	1.23	1.23	1.23	1.23	1.23	1.23	1.23
NaCl	0.30	0.30	0.30	0.30	0.30	0.30	0.30	0.30	0.30
Rice hull powder	0.40	0.40	0.40	0.40	0.40	0.40	0.40	0.40	0.40
Total	100.00	100.00	100.00	100.00	100.00	100.00	100.00	100.00	100.00
Nutritional levels ^3^									
Metabolizable energy MJ/kg	11.51	11.51	11.52	11.51	11.51	11.51	11.51	11.51	11.52
Crude protein, %	16.01	16.05	16.07	16.99	17.03	17.01	18.00	18.02	17.98
Lysine, %	0.68	0.85	0.98	0.70	0.83	0.97	0.67	0.85	0.98
Digestible lysine	0.61	0.76	0.91	0.62	0.74	0.90	0.61	0.75	0.90
Methionine, %	0.41	0.40	0.41	0.39	0.38	0.39	0.37	0.37	0.37
Digestible methionine	0.38	0.37	0.38	0.36	0.36	0.36	0.34	0.34	0.34
Methionine + Cystine, %	0.68	0.67	0.68	0.68	0.69	0.68	0.68	0.68	0.68
Digestible Methionine + Cystine	0.63	0.62	0.62	0.61	0.64	0.62	0.62	0.61	0.62
Calcium, %	0.85	0.86	0.85	0.85	0.85	0.86	0.84	0.85	0.85
Total phosphorus, %	0.66	0.65	0.65	0.68	0.66	0.65	0.68	0.67	0.65
Non-phytate phosphorus, %	0.40	0.40	0.40	0.41	0.40	0.40	0.41	0.41	0.40
Threonine, %	0.67	0.67	0.67	0.67	0.67	0.67	0.67	0.67	0.67
Digestible Threonine	0.59	0.58	0.58	0.58	0.58	0.58	0.58	0.58	0.58

^1^ Provided per kg of diet: Vitamin A, 6000 IU; Vitamin D, 2800 IU; Vitamin E, 20 IU; Vitamin K, 2.4 mg; Vitamin B12, 0.027 mg; Thiamine, 2.0 mg; Riboflavin, 5.2 mg; D-Calcium pantothenate, 11 mg; Niacin, 30 mg; Pyridoxine, 3.6 mg; Biotin, 0.2 mg; Folic acid, 1.2 mg; Choline, 800 mg; ^2^ Provided per kg of diet: Manganese, 90 mg; Iodine, 1.8 mg; Ferrous, 100 mg; Copper, 8 mg; Zinc, 80 mg; Selenium, 0.30 mg; ^3^ All nutrient levels are measured values except that ME and digestible amino acids were calculated values.

**Table 2:** Effects of dietary CP and lysine levels on growth performance at 9 to 16 weeks of age.

CP, %	Lysine, %	AFI, g	BWG, g	F/G (g:g)	Mortality Rate, %
16.0	0.69	480.80 ± 6.55 ^a^	87.28 ± 3.47	5.50	1.67 ± 3.73
16.0	0.84	470.34 ± 7.47 ^ab^	89.3 ± 7.65	5.27	3.33 ± 4.56
16.0	0.99	468.57 ± 9.22 ^abc^	88.92 ± 4.85	5.27	0
17.0	0.69	463.67 ± 9.63 ^bcd^	90.15 ± 4.46	5.14	6.67 ± 6.97
17.0	0.84	465.92 ± 5.24 ^bcd^	90.31 ± 8.42	5.16	0
17.0	0.99	480.25 ± 15.34 ^a^	89.87 ± 3.89	5.34	6.67 ± 3.73
18.0	0.69	453.08 ± 14.25 ^d^	90.85 ± 3.97	4.98	3.33 ± 4.56
18.0	0.84	462.81 ± 11.60 ^bcd^	94.85 ± 4.57	4.88	0
18.0	0.99	454.72 ± 3.52 ^cd^	96.27 ± 7.31	4.72	1.67 ± 3.73
Main effects					
CP, %	16.0	473.24 ± 9.15 ^a^	88.50 ± 5.26 ^b^	5.34	1.67 ± 3.45
	17.0	469.94 ± 12.63 ^a^	90.11 ± 5.50 ^ab^	5.22	4.44 ± 5.33
	18.0	456.87 ± 10.93 ^b^	93.99 ± 5.60 ^a^	4.86	1.67 ± 3.45
	*p*-value	<0.010	0.034	0.325	0.086
Lysine, %	0.69	465.85 ± 15.38	89.42 ± 4.02	5.21	3.89 ± 5.33
	0.84	466.36 ± 8.51	91.49 ± 7.01	5.09	1.11 ± 2.93
	0.99	467.85 ± 14.55	91.69 ± 6.14	5.10	2.78 ± 4.07
	*p*-value	0.850	0.488	0.329	0.150
CP × lysine	*p*-value	0.012	0.848	0.618	0.062

AFI = average feed intake; BWG = body weight gain; F/G = feed gain ratio; Data in the same column without letters or the same letters superscripts mean no significant difference (*p* > 0.05), different letters superscripts mean significant difference (*p* < 0.01 as high statistically significant, *p* < 0.05 as statistically significant, and 0.05 < *p* < 0.10 as having a significant trend.), the same below.

**Table 3:** Effects of dietary CP and lysine levels on slaughter performance at 9 to 16 weeks of age.

CP, %	Lysine, %	Dressed Percentage, %	Percentage of Half-Eviscerated Weight with Giblet, %	Percentage of Eviscerated Weight, %	Percentage of Breast Muscle Yield, %	Percentage of Leg Muscle Yield, %
16.0	0.69	90.51 ± 1.94	79.33 ± 1.64	68.88 ± 1.82	10.05 ± 0.68	10.89 ± 1.09 ^b^
16.0	0.84	89.63 ± 3.42	79.45 ± 1.90	70.38 ± 1.73	10.38 ± 1.53	12.66 ± 1.13 ^b^
16.0	0.99	90.51 ± 2.38	78.47 ± 2.06	68.73 ± 1.73	10.08 ± 0.50	11.77 ± 1.68 ^b^
17.0	0.69	90.05 ± 1.56	81.39 ± 2.30	72.81 ± 1.95	10.72 ± 0.39	13.46 ± 1.31 ^ab^
17.0	0.84	91.21 ± 3.19	81.63 ± 3.63	71.14 ± 3.79	10.41 ± 0.91	14.18 ± 1.30 ^a^
17.0	0.99	92.02 ± 1.10	80.59 ± 2.58	70.87 ± 3.22	10.85 ± 1.28	13.19 ± 1.53 ^ab^
18.0	0.69	92.19 ± 1.70	80.84 ± 1.92	72.27 ± 1.64	10.34 ± 0.62	13.99 ± 0.82 ^ab^
18.0	0.84	89.56 ± 2.97	78.15 ± 2.81	68.81 ± 2.85	10.36 ± 0.67	13.83 ± 1.02 ^ab^
18.0	0.99	91.88 ± 3.35	79.44 ± 2.93	70.58 ± 3.68	10.96 ± 0.56	13.74 ± 1.38 ^ab^
Main effects						
CP, %	16.0	90.22 ± 2.44	79.08 ± 1.76	69.33 ± 1.77	10.17 ± 0.96	11.77 ± 1.45 ^b^
	17.0	91.09 ± 2.11	81.2 ± 2.66	71.61 ± 2.93	10.66 ± 0.87	13.61 ± 1.33 ^a^
	18.0	91.21 ± 2.79	79.48 ± 2.61	70.55 ± 2.97	10.55 ± 0.81	13.86 ± 1.00 ^a^
	*p*-value	0.585	0.105	0.124	0.266	<0.01
Lysine, %	0.69	90.92 ± 1.84	80.52 ± 2.00	71.32 ± 2.44	10.37 ± 0.73	12.78 ± 1.91
	0.84	90.13 ± 3.00	79.74 ± 3.00	70.11 ± 2.83	10.38 ± 1.01	13.56 ± 1.35
	0.99	91.47 ± 2.33	79.5 ± 2.48	70.06 ± 2.88	10.63 ± 0.87	12.90 ± 1.64
	*p*-value	0.441	0.584	0.424	0.641	0.266
CP × lysine	*p*-value	0.663	0.739	0.433	0.733	0.595

Data in the same column without letters or the same letters superscripts mean no significant difference (*p* > 0.05), different letters superscripts mean significant difference (*p* < 0.01 as high statistically significant, *p* < 0.05 as statistically significant, and 0.05 < *p* < 0.10 as having a significant trend).

**Table 4:** Effects of dietary CP and lysine levels on meat quality at 9 to 16 weeks of age.

CP, %	Lysine, %	Drip Loss, %	Cooking Loss, %	pH_24h_	Shear Force, N
16.0	0.69	5.73 ± 1.24	16.17 ± 5.57	6.28 ± 0.19	20.84 ± 5.77
16.0	0.84	3.47 ± 0.55	15.32 ± 4.44	6.31 ± 0.11	21.64 ± 3.44
16.0	0.99	6.71 ± 2.95	17.44 ± 4.39	6.27 ± 0.06	19.91 ± 3.26
17.0	0.69	6.04 ± 1.62	16.70 ± 2.44	6.20 ± 0.17	22.73 ± 4.45
17.0	0.84	6.66 ± 2.11	17.36 ± 1.27	6.32 ± 0.13	24.48 ± 5.38
17.0	0.99	7.22 ± 1.10	16.83 ± 3.13	6.14 ± 0.08	23.41 ± 5.13
18.0	0.69	5.28 ± 1.20	15.63 ± 4.02	6.30 ± 0.12	28.66 ± 5.37
18.0	0.84	5.88 ± 5.37	14.27 ± 2.64	6.27 ± 0.21	25.74 ± 4.76
18.0	0.99	5.69 ± 1.74	16.05 ± 3.31	6.24 ± 0.10	34.26 ± 11.06
Main effects					
CP, %	16.0	5.30 ± 2.21	16.31 ± 4.46	6.28 ± 0.12	20.80 ± 4.28 ^b^
	17.0	6.64 ± 1.58	16.96 ± 2.20	6.22 ± 0.14	23.54 ± 4.95 ^b^
	18.0	5.62 ± 3.03	15.32 ± 3.15	6.27 ± 0.14	29.55 ± 8.26 ^a^
	*p*-value	0.378	0.550	0.502	<0.01
Lysine, %	0.69	5.68 ± 1.28	16.17 ± 3.84	6.26 ± 0.16	24.08 ± 6.12
	0.84	5.34 ± 3.34	15.65 ± 3.08	6.3 ± 0.14	23.96 ± 4.82
	0.99	6.54 ± 1.99	16.77 ± 3.36	6.22 ± 0.09	25.86 ± 9.43
	*p*-value	0.464	0.758	0.350	0.617
CP × lysine	*p*-value	0.592	0.949	0.757	0.329

Data in the same column without letters or the same letters superscripts mean no significant difference (*p* > 0.05), different letters superscripts mean significant difference (*p* < 0.01 as high statistically significant, *p* < 0.05 as statistically significant, and 0.05 < *p* < 0.10 as having a significant trend).

**Table 5:** Effects of dietary CP and lysine levels on myofiber characteristics of growing chickens.

CP, %	Lysine, %	Myofiber Density, (P·mm^−2^)	Myofiber Diameter, μm	Number of Fibers in the Muscle Bundle	Endomysium Thickness, μm	Perimysium Thickness, μm
16.0	0.69	862.13 ± 190.91 ^cd^	29.72 ± 7.02 ^d^	111.09 ± 49.58 ^ab^	5.03 ± 4.11 ^ab^	8.67 ± 5.48 ^bc^
16.0	0.84	1020.39 ± 103.47 ^ab^	27.28 ± 5.98 ^g^	109.88 ± 24.91 ^ab^	3.81 ± 1.46 ^d^	6.77 ± 3.12 ^de^
16.0	0.99	1092.6 ± 191.56 ^a^	24.29 ± 5.54 ^i^	100.64 ± 22.18 ^ab^	3.66 ± 1.41 ^d^	8.80 ± 3.41 ^bc^
17.0	0.69	1010.41 ± 116.69 ^ab^	25.99 ± 6.39 ^h^	91.52 ± 26.16 ^ab^	4.43 ± 1.54 ^c^	9.03 ± 3.40 ^b^
17.0	0.84	832.01 ± 193.59 ^cd^	27.96 ± 7.11 ^f^	105.64 ± 34.55 ^ab^	4.76 ± 1.71 ^b^	8.31 ± 3.34 ^c^
17.0	0.99	756.30 ± 111.71 ^d^	30.36 ± 6.90 ^c^	113.48 ± 25.11 ^a^	5.29 ± 1.71 ^a^	12.56 ± 3.96 ^a^
18.0	0.69	832.00 ± 187.93 ^cd^	30.75 ± 7.61 ^b^	109.45 ± 32.31 ^ab^	4.27 ± 1.35 ^c^	7.13 ± 2.41 ^d^
18.0	0.84	768.88 ± 159.58 ^d^	32.62 ± 8.15 ^a^	88.56 ± 14.59 ^b^	3.72 ± 0.96 ^d^	5.64 ± 2.39 ^f^
18.0	0.99	938.18 ± 120.55 ^bc^	29.40 ± 7.43 ^e^	107.92 ± 22.46 ^ab^	3.53 ± 1.21 ^d^	6.42 ± 3.86 ^e^
Main effects						
CP, %	16.0	991.70 ± 193.66 ^a^	27.10 ± 6.51 ^c^	107.20	4.16 ± 2.5 ^b^	8.08 ± 3.98 ^b^
	17.0	866.24 ± 176.76 ^b^	28.10 ± 7.04 ^b^	103.55	4.83 ± 1.7 ^a^	9.97 ± 4.05 ^a^
	18.0	846.35 ± 169.05 ^b^	30.92 ± 7.75 ^a^	101.98	3.84 ± 1.29 ^c^	6.40 ± 3.09 ^c^
	*p*-value	<0.01	<0.01	0.609	<0.01	<0.01
Lysine, %	0.69	901.51	28.82 ± 7.33 ^b^	104.02	4.58 ± 2.81 ^a^	8.28 ± 4.07 ^b^
	0.84	873.76	29.29 ± 7.17 ^a^	101.36	4.09 ± 1.58 ^c^	6.91 ± 3.25 ^c^
	0.99	929.03	28.02 ± 7.15 ^c^	107.35	4.16 ± 1.66 ^b^	9.26 ± 4.37 ^a^
	*p*-value	0.247	<0.01	0.569	<0.01	<0.01
CP × lysine	*p*-value	<0.01	<0.01	0.026	<0.01	<0.01

Data in the same column without letters or the same letters superscripts mean no significant difference (*p* > 0.05), different letters superscripts mean significant difference (*p* < 0.01 as high sta-tistically significant, *p* < 0.05 as statistically significant, and 0.05 < *p* < 0.10 as having a significant trend).

**Table 6:** Correlation between myofiber characteristics and meat quality indicators.

	Myofiber Density	Myofiber Diameter	Number of Fibers in the Muscle Bundle	Endomysium Thickness	Perimysium Thickness	Drip Loss	pH_24h_	Cooking Loss	Shear Force
Myofiber density	1.000	−0.883 **	−0.149	−0.523 **	−0.143	0.465	0.374	−0.057	−0.401
Myofiber diameter		1.000	0.084	0.448 **	−0.437 *	−0.384	−0.206	−0.262	0.455
Number of fibers in the muscle bundle			1.000	0.410	−0.353 *	0.379	0.238	−0.006	−0.066
Endomysium thickness				1.000	0.764 *	−0.123	−0.283	0.409	−0.306
Perimysium thickness					1.000	0.075	−0.272	0.553	−0.682 *
Drip loss						1.000	−0.527 **	0.546 **	0.078
pH_24h_							1.000	−0.834 **	−0.205
Cooking loss								1.000	0.380 *
Shear force									1.000

* means significant difference (*p* < 0.05); ** means very significant difference (*p* < 0.01).

## Data Availability

None of the data were deposited in an official repository. The data that support the study findings are available upon request.
